# Putative alternative translation start site-encoding nucleotides of *CPR5* regulate growth and resistance

**DOI:** 10.1186/s12870-020-02485-2

**Published:** 2020-06-29

**Authors:** Muhammad B. Faisal, Tsanko S. Gechev, Bernd Mueller-Roeber, Paul P. Dijkwel

**Affiliations:** 1 School of Fundamental Sciences, Massey University, Palmerston North, New Zealand; 2 Center of Plant Systems Biology and Biotechnology, 4000 Plovdiv, Bulgaria; 3 Department of Molecular Biology, Institute of Biochemistry and Biology, University of Potsdam, 14476 Potsdam, Germany

**Keywords:** CPR5, Plant growth, Disease resistance, Cell death, *Arabidopsis thaliana*, Endoreduplication

## Abstract

**Background:**

The Arabidopsis CONSTITUTIVE EXPRESSER of PATHOGENESIS-RELATED GENES 5 (CPR5) has recently been shown to play a role in gating as part of the nuclear pore complex (NPC). Mutations in *CPR5* cause multiple defects, including aberrant trichomes, reduced ploidy levels, reduced growth and enhanced resistance to bacterial and fungal pathogens. The pleiotropic nature of *cpr5* mutations implicates that the CPR5 protein affects multiple pathways. However, little is known about the structural features that allow CPR5 to affect the different pathways.

**Results:**

Our in silico studies suggest that in addition to three clusters of putative nuclear localization signals and four or five transmembrane domains, CPR5 contains two putative alternative translation start sites. To test the role of the methionine-encoding nucleotides implicated in those sites, *metCPR5* cDNAs, in which the relevant nucleotides were changed to encode glutamine, were fused to the *CPR5* native promoter and the constructs transformed to *cpr5–2* plants to complement c*pr5*-compromised phenotypes. The control and *metCPR5* constructs were able to complement all *cpr5* phenotypes, although the extent of complementation depended on the specific complementing plant lines. Remarkably, plants transformed with *metCPR5* constructs showed larger leaves and displayed reduced resistance when challenged to *Pseudomonas syringae* pv *Pst* DC3000, as compared to control plants. Thus, the methionine-encoding nucleotides regulate growth and resistance. We propose that structural features of the CPR5 N-terminus are implicated in selective gating of proteins involved in regulating the balance between growth and resistance.

**Conclusion:**

Plants need to carefully balance the amount of resources used for growth and resistance. The Arabidopsis CPR5 protein regulates plant growth and immunity. Here we show that N-terminal features of CPR5 are involved in the regulation of the balance between growth and resistance. These findings may benefit efforts to improve plant yield, while maintaining optimal levels of disease resistance.

## Background

Plants recognise attack of most pathogens and respond through the production of antimicrobial proteins and the activation of defence responses [1]. In an attempt to define the signalling processes leading to plant disease resistance and susceptibility, a variety of mutants were isolated that displayed a defective resistance response. For example, mutants with hyper-susceptibility phenotypes e.g., *npr1* for *non-expresser of pathogenesis related genes1* [2–4] and *eds5* for *enhanced disease susceptibility5* [5, 6] and mutants that show constitutive activation of defence-related genes and enhanced defence responses (e.g., *acd6* (*accelerated cell death6*), *cpr1* (*constitutive expresser of pathogenesis-related genes1*) [7], *cpr2* and *cpr6* [8]) were isolated. In 1997, Bowling and co-workers reported the *cpr5–2* mutant, which displayed constitutive expression of *pathogenesis-related genes PR1*, *PR5* and *PDF1.2*, aberrant trichomes and hypersensitive response (HR)-like lesions on their leaves in the absence of any pathogen attack [9]. Later, Jing et al. [10] described the *old1* (*onset ofleafdeath1*) mutant that showed HR-like lesions, early leaf senescence and the early onset of leaf death. In the same year, Yoshida et al. [11] reported *hys1* (*hypersenescence1)* as a mutant with accelerated leaf senescence and hypersensitivity to sugar. Borghi et al. [12] isolated *cpr5–3*, which showed reduced leaf K^+^ levels and *cpr5–2* phenotypes. *CPR5*, *OLD1* and *HYS1* were all found to be the same locus and subsequent studies showed that *cpr5* plants also exhibit hypersensitivity to exogenous abscisic acid (ABA), jasmonic acid (JA) and ethylene (ET) [13–15]. The pleiotropic nature of *cpr5* mutants suggests that CPR5 is a master regulator of different cellular pathways [16].

The functional analyses of the CPR5 protein have been difficult because this protein is only found in plants and the moss, *Physcomitrella patens* [13]. Nevertheless, computational studies showed that CPR5 is predicted to contain an N-terminal bipartite nuclear localisation signal (NLS) and multiple transmembrane (TM) domains at the carboxyl terminus [11], which is consistent with the model that CPR5 is a nuclear membrane protein [14]. Recently, CPR5 has emerged as one of the nucleoporins and shown to function as part of the nuclear pore complex (NPC) [14, 15, 17]. CPR5 was suggested to participate in NPC gating through the formation of CPR5-CPR5 homodimers and deregulated nucleocytoplasmic transport as a consequence of mutations in the *CPR5* gene was proposed to result in compromised immunity in *cpr5* mutants. However, it is not clear how structural features of the CPR5 proteins mediate selectivity.

In silico studies suggest that CPR5 contains three clusters of NLS-encoding residues, two clusters of Casein Kinase (CK) phosphorylation sites and a number of alternative translation start sites. Here, we mutated putative alternative translation start site-encoding residues in order to test their role in CPR5 function. The mutated genes were used to complement the *cpr5–2* mutant. We found that the complementing lines changed growth and disease resistance against *Pst* DC3000 demonstrating that the CPR5 N-terminus is involved in the regulation of plant growth and disease resistance.

## Results

### CPR5 is predicted to contain multiple translation initiation sites

**Fig. 1:**
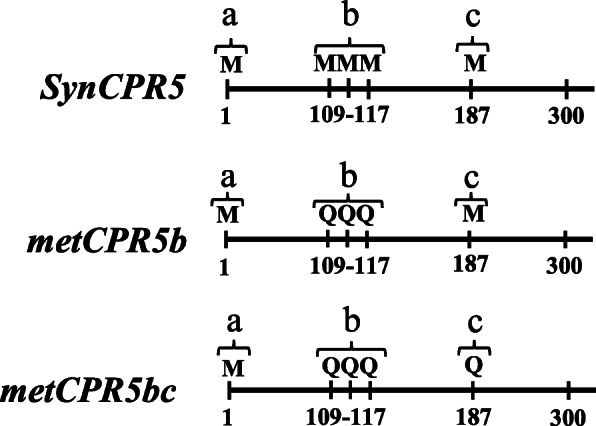
Schematic representation of *SynCPR5* and *metCPR5* transgenes. This figure shows the position of putative translation start site(s)-encoding residues in the *CPR5* synthetic gene. A synthetic *CPR5* gene was synthesised and is termed *SynCPR5*. Letters a, b and c indicate the predicted translation initiation sites whereas the numbers denote the position of nucleotides encoding methionine (M). The methionine-encoding nucleotides were substituted into CAA (glutamine, Q) in the *metCPR5b* and *metCPR5bc* synthetic genes

The *CPR5* transcript possess multiple in-frame start codons at positions a, b, and c as shown in Fig. 1. If these are functional alternative translation initiation sites then it would be expected that these sites contain features that favour translation initiation, such as stem-loop structures. Therefore, the *CPR5* mRNA sequence was further computationally analysed. Figure 1 shows the *CPR5* mRNA sequence and the positions of the in-frame start codons. A closer view of the second putative site (site b) shows that it consists of three in-tandem start codons, 109 nt downstream of the first start codon. The third site occurs 187 nt downstream of the first site. Furthermore, the *CPR5* coding sequence is predicted to contain four stem-loop structures in the first 190 nucleotides (Figure S1). Of these stem-loops, the first is present between the first and second putative start site. This stem-loop appears to be strong as 50% of the associated nuceleotides involved in the putative stem formation are guanine and cytosine. Notably, the second putative start site is predicted to be part of a stem-loop. Each of the putative translation start sites is followed by a stem-loop structure (Figure S1). Additionally, putative favourable residues for translation initiation such as adenine or guanine are also present at the expected positions (− 3, + 3 and + 4) of each putative initiation site (Figure S1). These results are consitent with the model that the *CPR5* transcript contains multiple translation initiation sites.

### Construction of a synthetic *CPR5* gene with mutated start codons

Complementation of *cpr5* mutants with the wild type *CPR5* gene rescues the compromised phenotypes associated with the mutation [18]. Therefore, we aimed to test the role of the putative alternative start sites in CPR5 function by complementing *cpr5–2* plants with synthetic *CPR5* constructs carrying mutations in the putative sites. In order to distinguish the complementing transgenic transcripts from *cpr5–2* transcripts, a number of nucleotide changes were introduced in the synthetic *CPR5* coding sequence by codon optimization. The codon optimized synthetic version of the *CPR5* gene was termed *SynCPR5* (Figs. 1 and S2) and constructs in which the putative start sites were mutated individually as well as collectively were called *metCPR5b* and *metCPR5bc*. The *metCPR5b* RNA translates into a CPR5 protein with the three methionines of the second putative start site converted into glutamine residues. Glutamine was chosen for substitution since it resembles methionine in size and is presumed to have limited impact on protein tertiary structure [19]. The *metCPR5bc* RNA has the third putative start site mutated in addition to the second (Fig. 1). Thus, these synthetic constructs will allow the separate detection of endogenous *cpr5–2* mRNA from the mRNA derived from the synthetic constructs in *cpr5–2* lines complemented with the synthetic *CPR5* genes.

### *CPR5* native promoter is able to drive *SynCPR5* expression

**Fig. 2:**
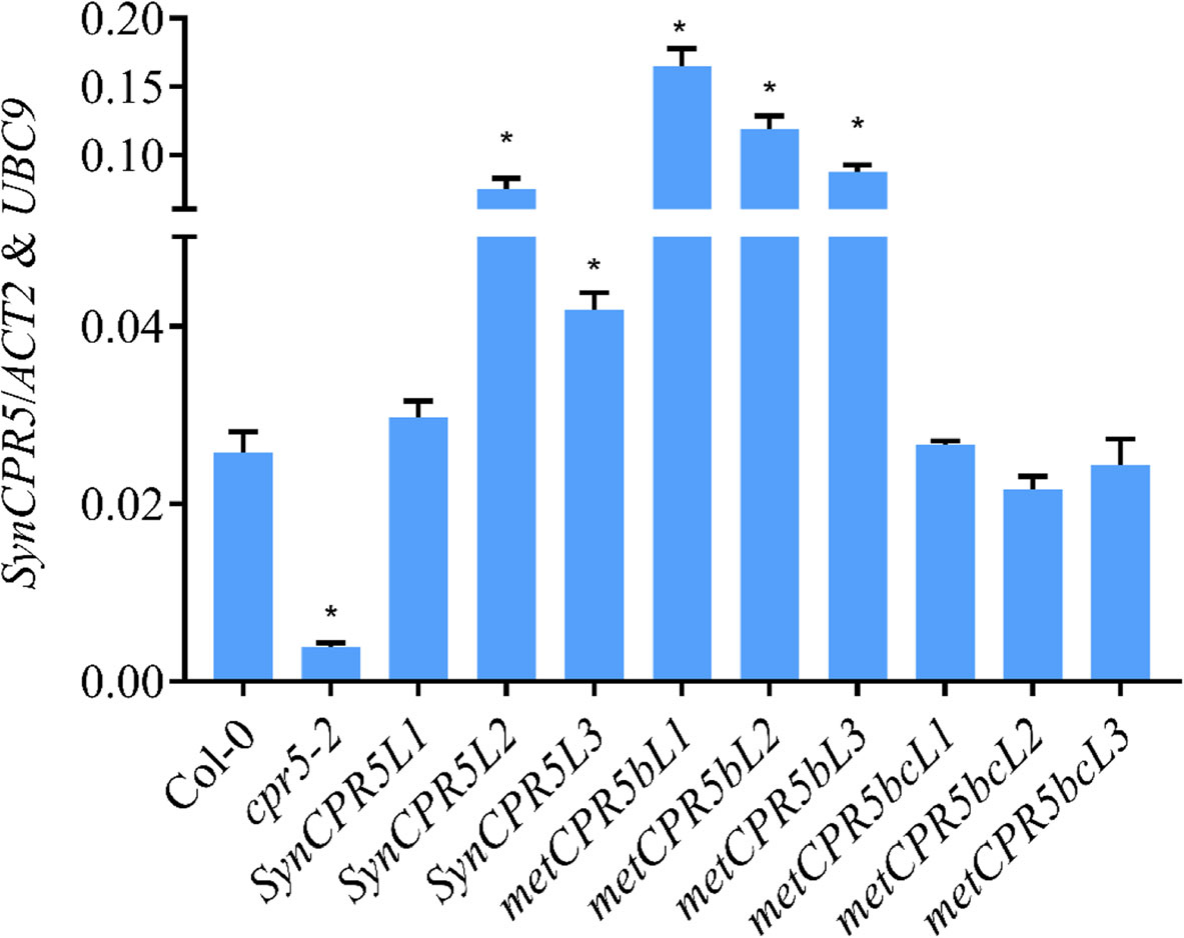
Quantification of *SynCPR5* transcripts in *SynCPR5* and *metCPR5* plants. Quantification of transcript abundance in *SynCPR5*, Col-0 and *cpr5–2* plants using *SynCPR5*, *CPR5* and *cpr5–2* specific sets of primers. Three biological and three technical replicates were included, and values were normalized to the average expression values of *ACT2* and *UBC9* housekeeping genes. Error bars represent the standard error, and asterisks indicate significant difference from Col-0 at *p* < 0.05 (Student’s *t*-test)

A number of studies have used the *Cauliflower Mosaic Virus 35S* promoter to drive expression of the *CPR5* gene [13, 20, 21]. Here, we expressed *SynCPR5* from the native *CPR5* promoter to mimic expression of the wild type *CPR5* gene. Actual levels of *SynCPR5* transcripts in transgenic lines were, however, expected to be lower than in the wild type, wild-type-like or higher than in the wild-type due to position effects [22, 23]. A 1.5-kb upstream fragment of the *CPR5* gene (AT5G64930) was fused with the *SynCPR5* coding sequence, and the whole cassette was transferred into *cpr5–2* plants. Following successful *SynCPR5* transformation, *SynCPR5* expression of three independent homozygous complemented lines was compared with *CPR5* expression in the wild type Col-0 and mutant *cpr5–2* lines. Relative transcript abundance of *SynCPR5*, *CPR5* and *cpr5–2* genes was quantified using *SynCPR5*, *CPR5* and *cpr5–2* gene-specific primer sets (Figure S2 and Table S1), as described in Methods. As shown in Fig. 2, the relative *SynCPR5* transcript abundance varied between the independent *SynCPR5* lines. *SynCPR5L1* showed wild type-like expression, whereas *SynCPR5L2* and *SynCPR5L3* displayed *SynCPR5* transcript abundance higher than the wild type. To summarise, these results establish that the 1.5-kb native *CPR5* promoter was able to drive *SynCPR5* expression in transgenic Arabidopsis plants.

### Transgenic *metCPR5* lines exhibit bigger leaves than *SynCPR5* and wild type lines

**Fig. 3:**
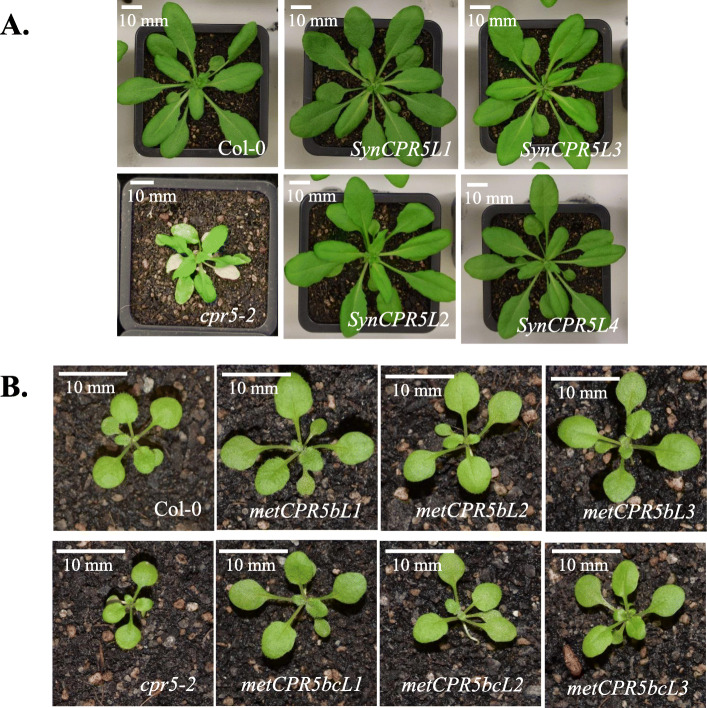
Growth and development of Col-0, *cpr5–2*, *SynCPR5* and *metCPR5* plants. Plants were grown in soil under long-day conditions and grown for 31 (**a**) or 17 (**b**) days. Representative plants were photographed. Photographs were cropped and resized with constant aspect ratio; bar indicates 10 mm

**Fig. 4:**
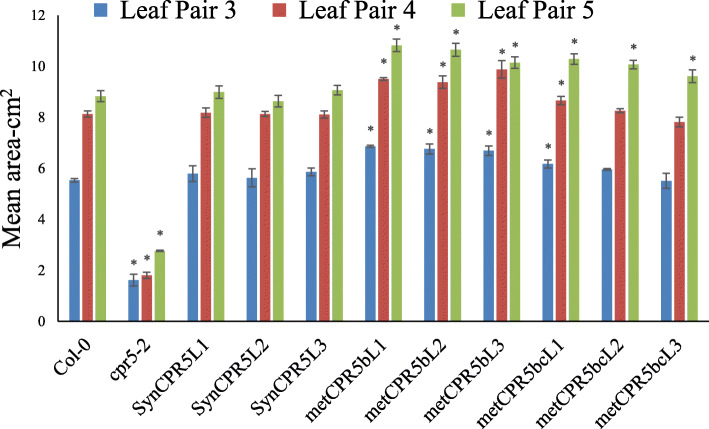
Mean leaf area of rosette leaf pairs. Mean leaf areas of third, fourth and fifth rosette leaf pairs at 31 DAS. The leaves were removed from five different plants of the same line and leaf area was measured as described in Methods. Error bars represent the standard error, and asterisks indicate significant difference from Col-0 at *p* < 0.05 (Student’s *t*-test)

Next, the *metCPR5b* and *metCPR5bc* constructs were transferred to *cpr5–2* plants to determine the effect of mutated putative translation initiation sites on *CPR5* activity. Three independent homozygous lines for each construct were selected for analysis and relative *metCPR5* levels measured. As shown in Fig. 2, all *metCPR5b* lines displayed higher mRNA levels than wild type, while *metCPR5bc* lines showed wild type-like (Col-0) transcript abundances. During growth of the different transgenic and control wild-type and *cpr5–2* plant lines grown under same conditions, we noted that the leaves of *metCPR5* plants appeared to be bigger than those of the wild type (Fig. 3). This prompted us to quantify leaf sizes of the different plant lines. Plants were grown for 31 days at standard growth conditions and the leaf sizes of the third to fifth leaf pairs were measured as described in Methods. As shown in Figs. 3 and 4, the leaf areas of the *SynCPR5* lines were indifferent from those of the wild type. In contrast, the leaf areas of the third (fifth and sixth rosette leaves), fourth (seventh and eighth rosette leaves) and fifth (ninth and tenth rosette leaves) leaf pairs of *metCPR5b* plants were significantly larger than those of wild-type and *SynCPR5* lines (Fig. 4). Furthermore, the leaf areas of the fifth leaf pair of *metCPR5bc* plants were larger than those of wild type and *SynCPR5* plants. In addition to leaf area, we determined the sizes of epidermal pavement cells. The cells of *metCPR5bc* plants were significantly bigger than those of the wild type (*p* < 0.05; Student’s *t*-test) (Figure S3). Thus, these results indicate that the second putative alternative translation start site restricts leaf size.

### *metCPR5b* and *metCPR5bc* leaves have higher ploidy levels than wild type

**Fig. 5:**
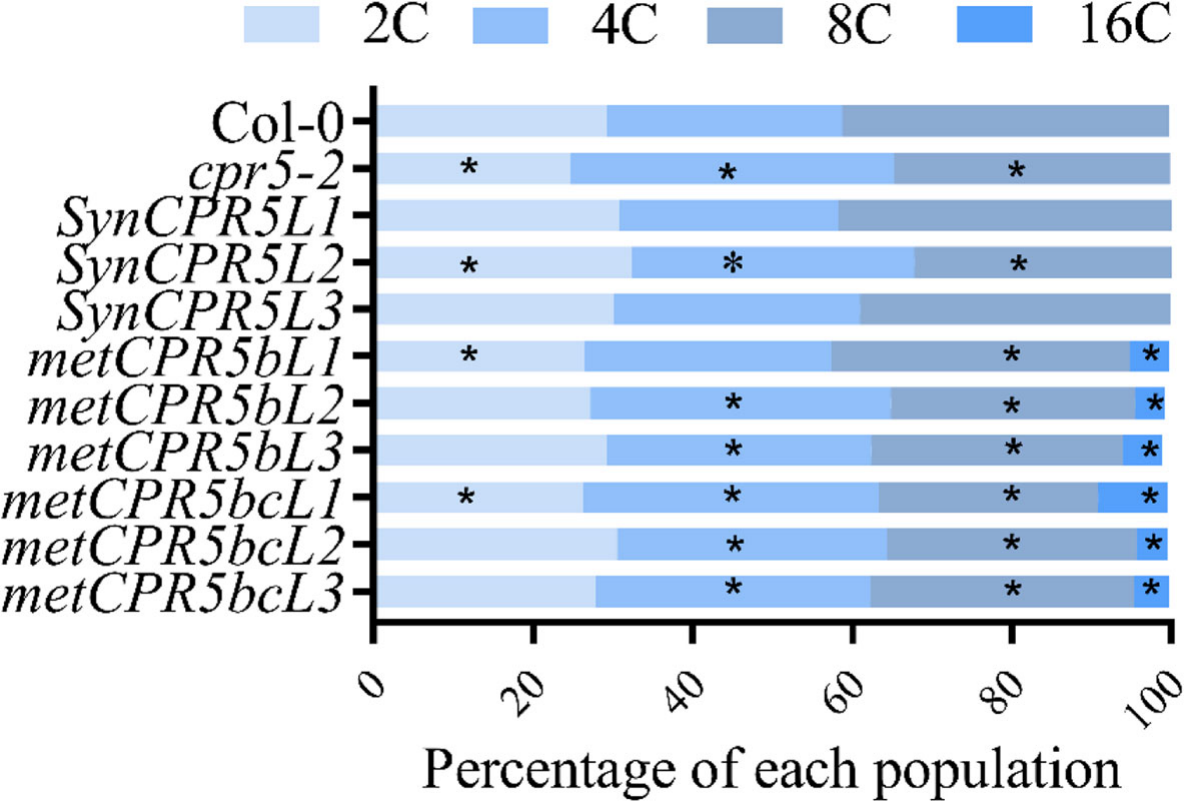
Ploidy levels of nuclei isolated from leaf tissue. Figure shows percentages of nuclei populations extracted from leaf cells of Col-0, *cpr5–2*, *SynCPR5* and *metCPR5* plants. The nuclei were extracted from leaf cells of 24-old-plants and analysed by flow cytometry as described in Methods. Percentages of every population from each sample were calculated and average (percentage) values are shown in the graph. Each value represents the mean of three values from leaves of three different plants of the same line. Asterisks indicate significant difference from Col-0 at *p* < 0.05 (Student’s *t*-test)

Leaf size is positively correlated with ploidy levels [24]. In *A. thaliana*, leaves generally undergo three to four rounds of endoreduplication and consequently contain nuclear populations of DNA content up to 16 or 32 C [24]. We hypothesised that *metCPR5* lines, having bigger leaves, have higher ploidy levels than wild type. To test this hypothesis, the third and the fourth leaves from 24-day-old (24 DAS) *metCPR5*, *SynCPR5* and wild type plants were harvested and nuclei were extracted for ploidy analysis (Fig. 5). Flow cytometry of the isolated nuclei showed that two of the *SynCPR5* lines had ploidy levels indifferent from the wild type, while one had higher 2C and 4C levels, but lower 8C levels. Most *metCPR5* lines had similar 2C, but higher 4C levels than the wild type. Interestingly, all *metCPR5* lines displayed lower 8C levels and in addition, a 16C population was found (Fig. 5), while 16C nuclei were not detected in the other lines. These results support the hypothesis that the enlarged leaves are caused by increased ploidy levels in *metCPR5* lines.

### *metCPR5* plants conferred enhanced susceptibility to *P. syringae*

**Fig. 6:**
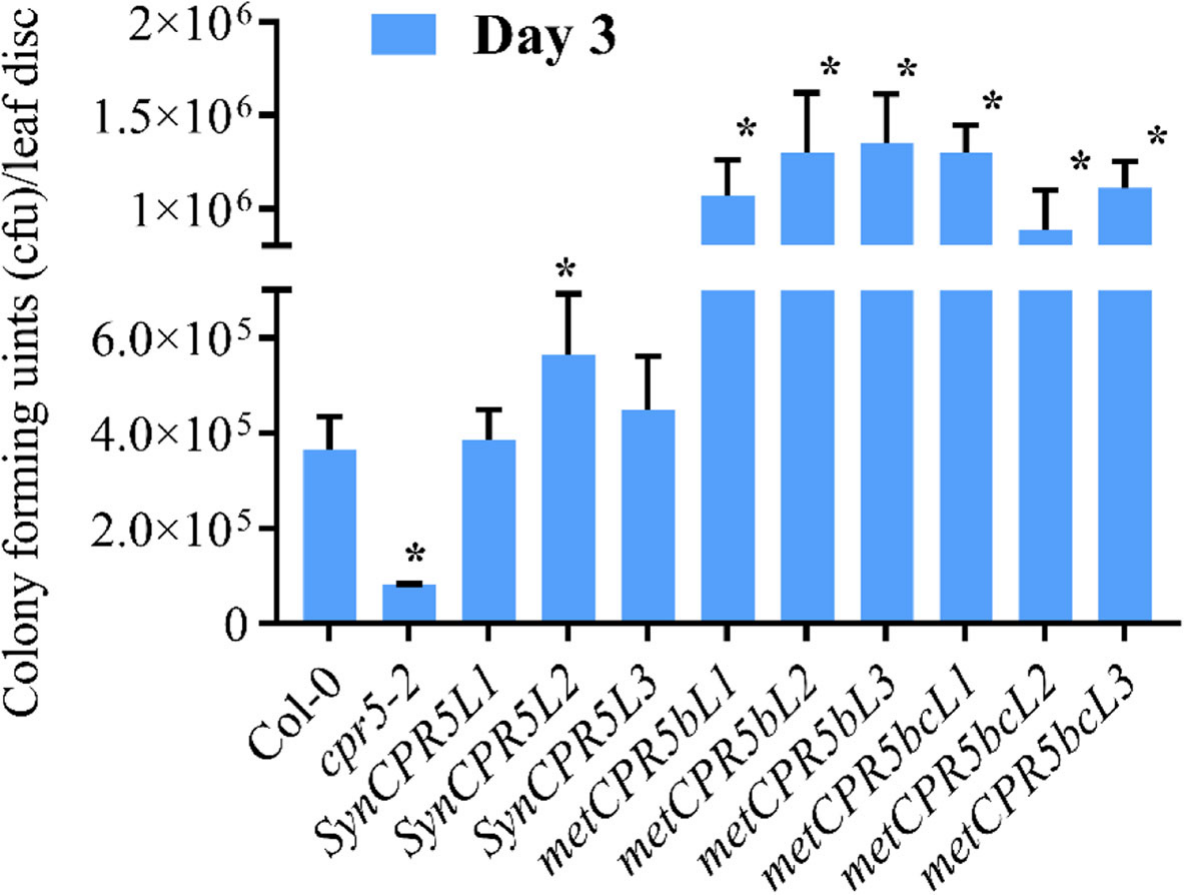
*Pst* DC3000 infection growth assay. Three leaf discs were cut from randomly selected leaves of three plants that were inoculated with *Pst* DC3000 and bacteria were extracted as described in [25]. *Pst* DC3000 bacteria were extracted from leaf discs (1 cm^2^) and plated onto agar plates. Colony forming units (cfu) were measured after 3 days of growth. The error bars represent the standard error of the mean and asterisks indicate significant difference from Col-0 at *p* < 0.05 (Student’s *t*-test)

Since *cpr5–2* mutants display reduced leaf size combined with enhanced resistance to infection from *Pseudomonas syringae* pv. *tomato* DC3000 (*Pst* DC3000), we hypothesised that the larger leaves of *metCPR5* plants may coincide with increased susceptibility to *Pst* DC3000. Thus, *metCPR5* plants were grown in neutral days (12:12 light:dark) conditions. Leaves from 4- to 5-week-old plants were infiltrated with *Pst* DC3000, and susceptibility was scored by measuring the number of colony forming units (cfu) as described (52). As shown in Fig. 6, the *metCPR5b* and *metCPR5bc* lines displayed significantly higher susceptibility to *Pst* DC3000 compared to the wild type. In contrast, *SynCPR5* lines displayed susceptibility to *Pst* DC3000 in a wild type manner except *SynCPR5L2*, which displayed higher susceptibility than the wild type. In conclusion, *metCPR5* plants, having larger leaves than wild type, show reduced resistance to *Pst* DC3000 infection.

### *metCPR5* lines have variable *PR1* levels

**Fig. 7:**
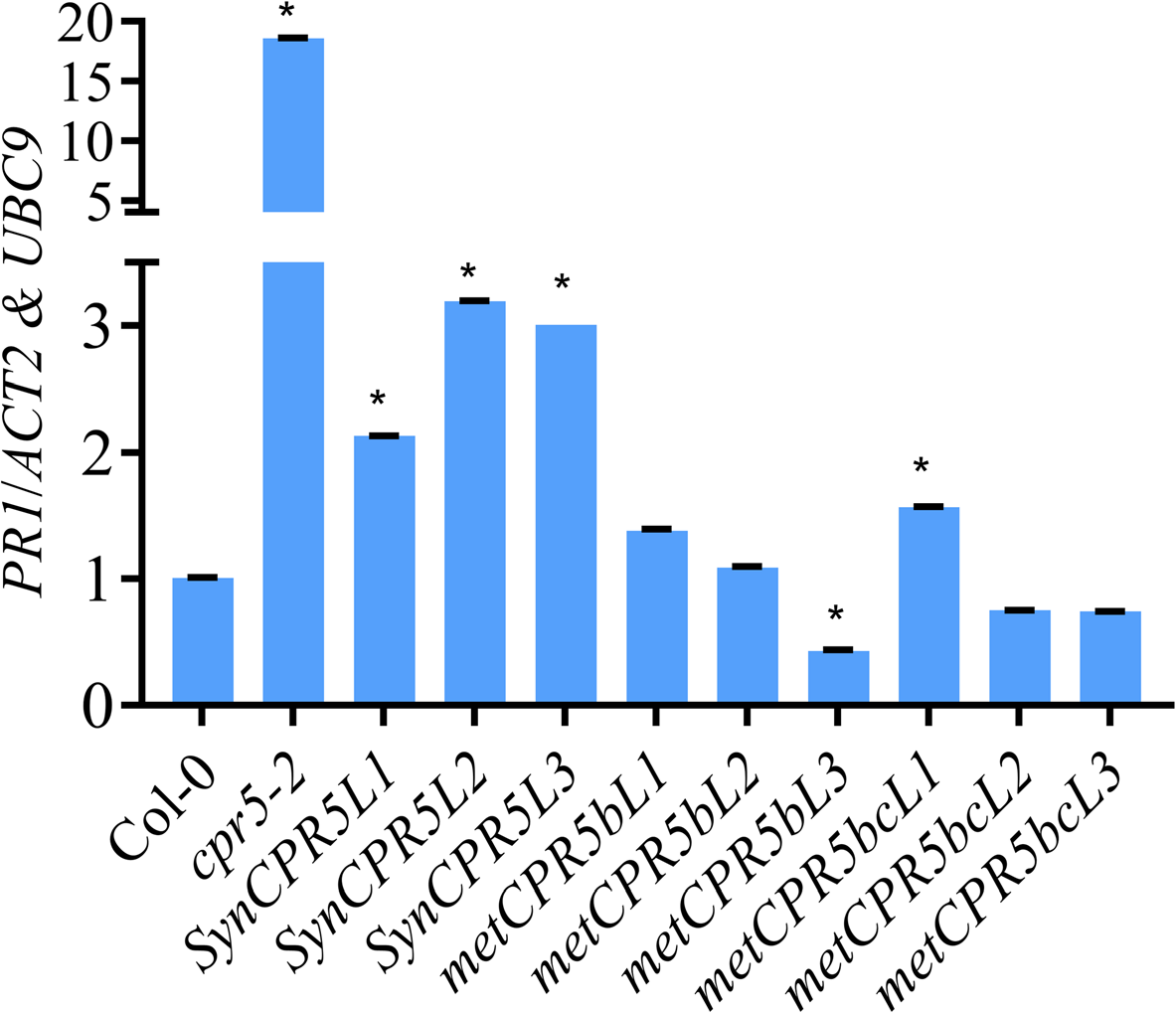
Relative expression levels of *PR1* in *SynCPR5* plants. Total RNA was extracted from whole rosettes of 31-day-old plants, and relative levels of *PR1* mRNA were measured. Three biological and three technical replicates were included, and values were normalized to the average expression values of *ACT2* and *UBC9* housekeeping genes. Error bars represent the standard error of the mean and asterisks indicate significant difference from Col-0 at *p* < 0.05 (Student’s *t*-test)

Expression of SA-mediated disease resistance marker gene *PATHOGENESIS RELATED GENE1* (*PR1*) is enhanced in *cpr5–2* mutants and correlates with resistance [9]. Therefore, it was expected that *metCPR5* lines have reduced *PR1* levels. The transcript abundance of *PR1* was quantified from uninfected wild type, *cpr5–2*, *SynCPR5* and *metCPR5* lines using real-time qRT-PCR. As shown in Fig. 7, *cpr5–2* plants showed greatly increased *PR1* levels. However, most *metCPR5* lines displayed *PR1* transcript levels similar to those of the wild type. The exceptions were *metCPR5bL3* and *metCPR5bcL1* lines, which had lower and higher *PR1* levels, respectively. Remarkably, all *SynCPR5* lines exhibited increased *PR1* levels compared to wild type (Fig. 7).

## Discussion

### Role of CPR5 in growth and resistance regulation

Plants balance their resource allocation between resistance and growth to limit negative impacts on plant growth [26, 27]. To further minimise cost, plants have developed distinct, highly orchestrated defense systems for different types of biotic and abiotic stresses [29–30]. For example, salicylic acid (SA)-mediated resistance is activated in response to biotrophic pathogens, whereas JA/ET-mediated resistance is induced upon necrotrophic pathogen invasion [31]. The constitutive activation of defense systems results in slow or reduced growth and development [28, 29, 32]. The phenotype of the *cpr5* mutant, i.e. compromised growth combined with increased resistance against the pathogens *P. syringae* and *Hyaloperonospora parasitica* [9] is consistent with this notion and suggests that resistance in *cpr5* is gained at the cost of growth. This is also in agreement with the finding that a large number of genes involved in auxin and gibberellin (GA) biosynthesis and signalling are repressed in *cpr5* [17]. Perhaps the simplest explanation of the phenotype is that CPR5 functions as a suppressor of resistance. A number of observations suggest that this is the case: the upregulation of *Pathogenesis-Related * genes (*PR1* and *PR5*) and *Plant Defensin 1.2* (*PDF1.2*), the formation of HR-like lesions and the higher SA levels in *cpr5* plants [8, 9, 11, 14]. Furthermore, the deregulation of cellular redox balance in *cpr5* plants has been proposed to be involved in the induction of lesions and early leaf senescence [33], suggesting that CPR5 may supress resistance by regulating ROS levels [16]. Similarly, the hypersensitive response of *cpr5* plants to the hormones SA, JA, ABA and ET [13, 20] indicates that CPR5 may regulate SA- and JA/ET-mediated resistance by suppressing the magnitude of these hormone responses. CPR5 has also been shown to suppress a set of genes involved in the biosynthesis of the cell wall [21], which functions as a first barrier against pathogen infection [34]. Nevertheless, aberrant trichomes, smaller epidermal cells and reduced ploidy levels clearly show that CPR5 is involved in growth regulation as well and it has been suggested that activation of resistance in *cpr5* could be the consequence of growth defects [35], including abnormal cell wall synthesis [36].

We show here that nucleotide changes in *metCPR5* result in plants that grow larger leaves than the wild type, at the expense of resistance to *Pst* DC3000. The mutated base pairs, rather than mRNA abundance, appear to be critical because the phenotypes correlated with the type of mutations and not the mRNA abundance. This is also consistent with previous results where overexpression of the wild type *CPR5* gene using the 35S promoter resulted in 1000-fold increase in gene expression, without similar larger leaves and decreased resistance being reported [13]. This suggests that the observed phenotype is a result of structural properties of the metCPR5 protein. However, we cannot exclude the possibility that the methionine-encoding nucleotides somehow limit protein translation which is overcome by the *metCPR5* mutations, leading to higher protein levels. The results are furthermore consistent with the model that CPR5 does not simply function as a repressor of disease resistance, but it regulates the balance between growth and resistance, whereby structural properties - present at the N-terminus - limit the extent by which CPR5 can favour growth over resistance.

### How does CPR5 control the growth-resistance balance?

The mechanism of how plants regulate the balance between growth and resistance is poorly understood due to its complex nature and involvement of a large number of growth- and resistance-related genes or hormones. For example, auxins, brassinosteroids, cytokinins, gibberellins, abscisic acid, ethylene, and jasmonates, have all been shown to be involved in the regulation of plant growth and immunity [26, 37–43]. Notably, the levels or signalling pathways of many of these hormones are affected in *cpr5* mutants as well: the levels of salicylic acid (SA) and jasmonic acid (JA) are higher in *cpr5* plants, *cpr5* plants have perturbations in SA, JA, ABA and ET signalling pathways [13], and *cpr5* plants are hypersensitive to exogenous SA, ABA, JA and ET [13, 15]. Recently, Meng et al. (2017) showed that CPR5 regulates growth and stress response-tradeoff through the modulation of SA and the unfolded protein response. In addition, a large number of growth promoting genes such as auxin and GA response genes are suppressed in *cpr5* mutants [17]. Nuclear accumulation of DELLA proteins leads to the repression on GA signalling, whereas their degradation release repression on GA biosynthesis signalling [44]. The misregulation of DELLA target genes in *cpr5* plants indicates that GA levels could be affected in *cpr5* plants as well [17]. Taken together, these results indicate that altered levels or signalling of the abovementioned hormones may affect growth and resistance in *cpr5* mutant plants. This is in line with our hypothesis that CPR5 plays a central role in regulating the balance between growth and resistance by mediating growth and resistance hormones. Recent discoveries of how CPR5 functions provides a means to propose how CPR5 controls growth and resistance: CPR5 is one of the nucleoporins which function as part of the Nuclear Pore Complex (NPC) and inhibit the activities of NPC and the cyclin-dependent kinases (CKIs) under stress-free conditions [14, 15, 17]. As a consequence of conformational changes in *cpr5* mutants or as a result of stress, CPR5-mediated gating is altered, allowing a number of molecules to enter the nucleus, leading to the activation of resistance genes [17]. Furthermore, CPR5 participates in NPC gating and acts as a selective barrier through the formation of CPR5-CPR5 homodimers [17]. The N-terminal region of CPR5 is crucial for CPR5-CPR5 homomeric interaction within the NPC and when this interaction was lost in the *old1-l3* allele of *CPR5* (G120D), proteins such as NPR1, JAZ1, and ABI5 were found to be aberrantly not retained in the cytoplasm [17]. These results are in general agreement with ours, which show that mutations that cause changes in the N-terminal part of the CPR5 protein can improve plant growth at the expense of disease resistance, possibly through altered CPR5-CPR5 homodimerization and CPR5-mediated NPC gating. Nevertheless, in addition to possible structural changes of metCPR5 proteins, the nucleotide changes in *metCPR5* transcripts may affect CPR5 function in other ways: the mutations remove possible alternative translation initiation sites and different alternatively translated CPR5 proteins may affect NPC gating. Indeed, in silico analysis of the *CPR5* transcript identified potential RNA secondary structures nearby putative alternative translation start sites and similar secondary structures have been shown to be important for the activity of alternative translation start sites [45–49]. Alternatively, or in addition, the induced mutations may have affected the secondary structures themselves and as such may have modified translation.

## Conclusion

Taken altogether, these results led us to propose that the changed nucleotides in *metCPR5* could have altered CPR5-CPR5 homodimerization and consequently CPR5-mediated NPC gating. Any structural difference(s) in the NPC complex could then affect the balance between growth and resistance by a changed gating of growth and/or resistance regulators.

## Methods

### Plant materials and growth conditions

*Arabidopsis thaliana* Columbia (CPR5 and *cpr5–2*) seeds were the same as used in [50] studies. Seeds were washed and disinfected according to the method described by [51] and imbibed in the dark for 3–4 days at 4 °C. Imbibed seeds were grown on autoclaved Daltons Premium Seed Raising Mix®, New Zealand. Unless mentioned, the plants were grown at 22 °C and 65% Relative Humidity (RH) under long-day conditions (16 h light, 8 h dark). For *Pseudomonas syringae* bacterial infiltrations, plants were grown under short-day conditions (10 h light, 14 h dark) at 21 °C and 65% RH.

### Trichome counting, leaf and pavement cell area measurements

Leaves were removed from plants grown and mounted on a glass slide. Number of appendages on each trichome was counted under a dissecting microscope. At 31 DAS (days after sowing), leaf pairs were removed from plants and fed onto a leaf area machine (LI-3050A/4, LI-COR, USA) for the combined measurement of both leaves. Leaves were harvested in pairs: leaf 1 and 2 were considered as leaf pair 1, leaf 3 and 4 as leaf pair 2, and so on. Leaf 3 from three different plants of the same transgenic line was imaged using a scanning electron microscope (FEI Quanta 200 Environmental Scanning Electron Microscope (SEM) with EDAX module) following manufacturer’s instructions at Manawatu Microscopy Imaging Centre (MMIC), Massey University, Palmerston North. The images were processed and cell areas were measured individually by ImageJ (https://imagej.nih.gov/ij/).

### *CPR5* gene synthesis and transformations

The modified *CPR5* gene variants, *SynCPR5* (Figure S1) and *metCPR5*, were synthesized by GenScript (GenScript®, USA). The *CPR5* gene variants, along with a 1.5 kb native *CPR5* promoter fragment, were cloned into pGreen0229 by GenScript. Sequence fidelity of cloned *SynCPR5* and *metCPR5* constructs was confirmed by Sanger sequencing and restriction digestion analyses. Plasmids were transformed into *Agrobacterium tumefaciens GV3101*, which was then used to transform *cpr5–2* plants using floral dipping as described [52]. Seeds harvested from the first generation of the transformed plants were grown on soil beds for screening of positive transformants using BASTA (125 mg/L). Homozygous transformed lines were selected in subsequent generations. The presence of the transgene in transformed lines was confirmed by DNA sequencing, restriction digestion analyses and PCR using *CPR5*, *SynCPR5* and *metCPR5* transgene-specific sets of primers (Figure S1).

### Ploidy level measurements, qRT-PCR data analyses and pathogenicity assays

For flow cytometry, nuclei were isolated, stained and analysed as described [21]. Kinematic analyses (DNA content measurements) of the *CPR5* transgenic lines, including Col-0 (CPR5) and *cpr5–2*, were carried out using a Sysmec Cyflow cytometer (Partec, CyFlow®, USA) as per manufacturer’s instructions. Total RNA was isolated from plants using Quick-RNA Miniprep kit (Zymo Research) and cDNA was prepared using Transcriptor First Strand cDNA Synthesis Kit (Roche). Transcript abundance was quantified using the LightCycler 480 Real-Time PCR (Roche) system [25]. Transcript abundance and statistical analyses were performed using Microsoft Office Excel 2010 as described [53]. *Pseudomonas syringae* pv DC3000 pathogenicity assays were carried out as described [25].

## Supplementary information

**Additional file 1: Figure S1:** Positions of putative translation start sites and RNA stem-loop structures. **Figure S2:** Sequence comparison and positions of primers used for real-time qRT-PCR quantifications. **Figure S3:** Area of abaxial epidermal pavement cells. **Table S1:** List of primers.

## Data Availability

All data generated and/or analysed during this study are included in this published article and its supplementary information files. The datasets used and/or analysed during the current study are available from the corresponding author on reasonable request.

## Abbreviations

ABA: Abscisic acid
*ABI5*: *ABA Insensitive 5*
*acd6*: *Accelerated cell death 6*
*AT3G18780*: *ACTIN 2*
*AT4G27960*: *Ubiquitin Conjugating Enzyme 9* (*UBC9*)
*AT5G64930*: *CPR5*
cDNA: Complementary DNA
CK: Casein kinase
CKIs: Cyclin-dependent kinases
*cpr1*: *Constitutive expresser of pathogenesis-related genes 1*
*cpr2* and *cpr6*: *constitutive expresser of pathogenesis-related genes 1* and *6*
*CPR5*: *CONSTITUTIVE EXPRESSER of PATHOGENESIS-RELATED GENES 5*
DAS: Days after sowing
*eds5*: *Enhanced disease susceptibility 5*
ET: Ethylene
*hys1*: *Hypersenescence 1*
JA: Jasmonic acid
JAZ1: Jasmonate ZIM-domain 1
NLS: Nuclear localisation signal
NPC: Nuclear pore complex
*npr1*: *Non-expresser of pathogenesis-related genes 1*
*NPR1*: *NON-EXPRESSER OF PATHOGENESIS-RELATED GENES 1*
*old1*: On*set of leaf death 1*
*PDF1.2*: *PLANT DEFENSIN 1.2*
*PR1*: *PATHOGENESIS RELATED GENE 1*
RNA/DNA: Ribonucleic acid / deoxyribonucleic acid
SA: Salicylic acid
SynCPR5: Synthetic version of CPR5
TM domains: Transmembrane domains
