# Supplementary material for: Putative alternative translation start site-encoding nucleotides of CPR5 regulate growth and resistance
Source: BMC Plant Biol. 2020 Jun 29;20:295. doi: 10.1186/s12870-020-02485-2 (PMC7322872; doi:10.1186/s12870-020-02485-2)
Supplement: Supplementary file 1 — Additional file 1: Figure S1: Positions of putative translation start sites and RNA stem-loop structures. Figure S2: Sequence comparison and positions of primers used for real-time qRT-PCR quantifications. Figure S3: Area of abaxial epidermal pavement cells. Table S1: List of primers. [file 12870_2020_2485_MOESM1_ESM.docx]

**Putative start site B**

**Putative start site C**

**Putative start site A**

Figure S1 Positions of putative translation start sites and RNA stem-loop structures

Positions of the predicted stem-loop structures in the *CPR5* coding sequence. The nucleotides highlighted in red boxes represent putative translation start sites (ATG), and the residues in green oval represent the position of putative nucleotides, which favour translation initiation from the putative site.

**
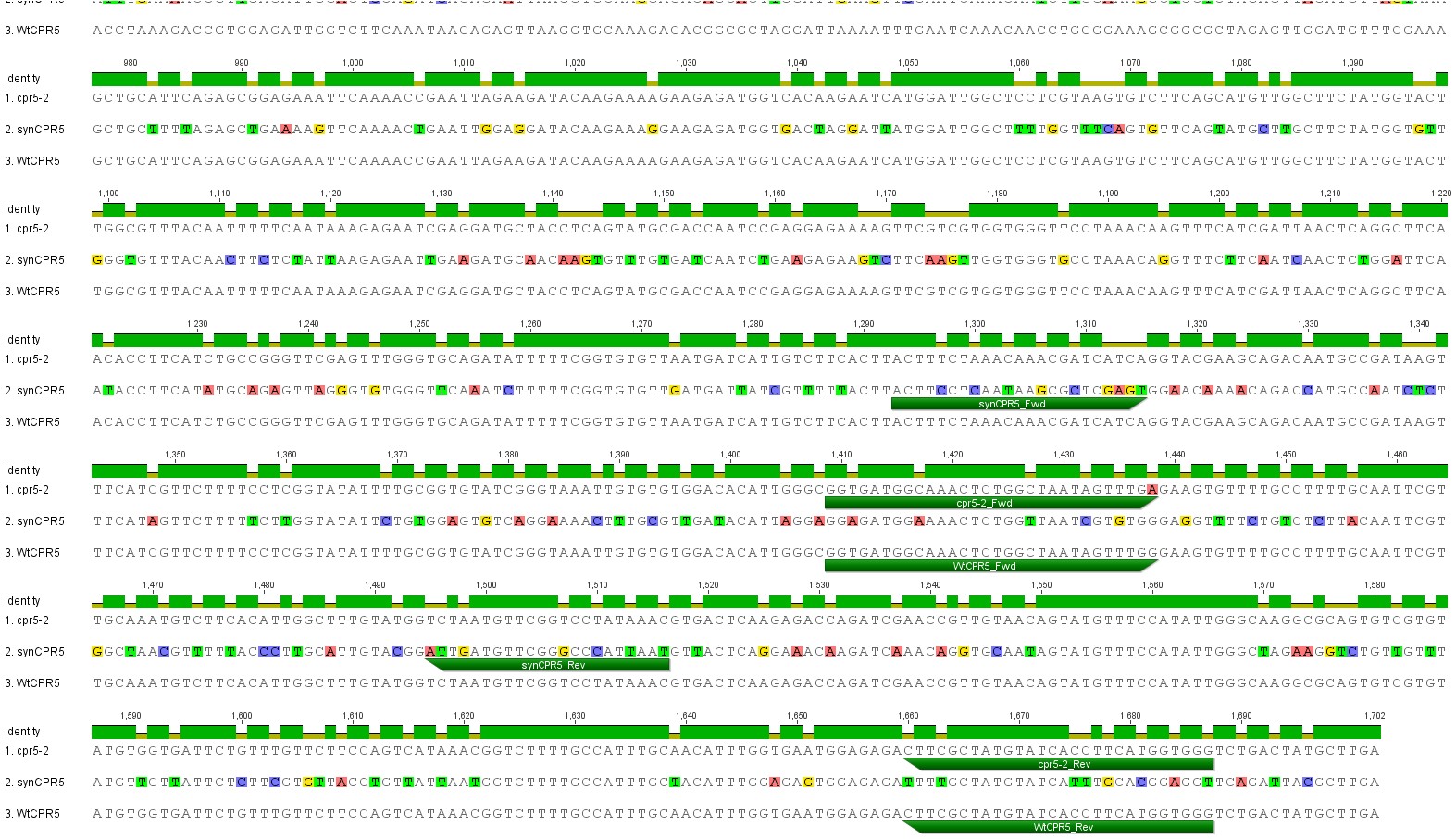
**

Figure S2 Sequence comparison and positions of primers used for real-time qRT-PCR quantifications

Sequence homology between *CPR5* (WtCPR5), *cpr5* mutant and *SynCPR5* (synthetic version) and the positions of the primer sets (sense and antisense) used for the quantification of transcript abundance of *CPR5* (wildtype), *cpr5-2* (mutant) and *SynCPR5* (synthetic) genes. Figure was constructed by aligning nucleotide sequences of *CPR5* (wildtype), *cpr5-2* (mutant) and *SynCPR5* (synthetic) genes using Geneious (<https://www.geneious.com/>).


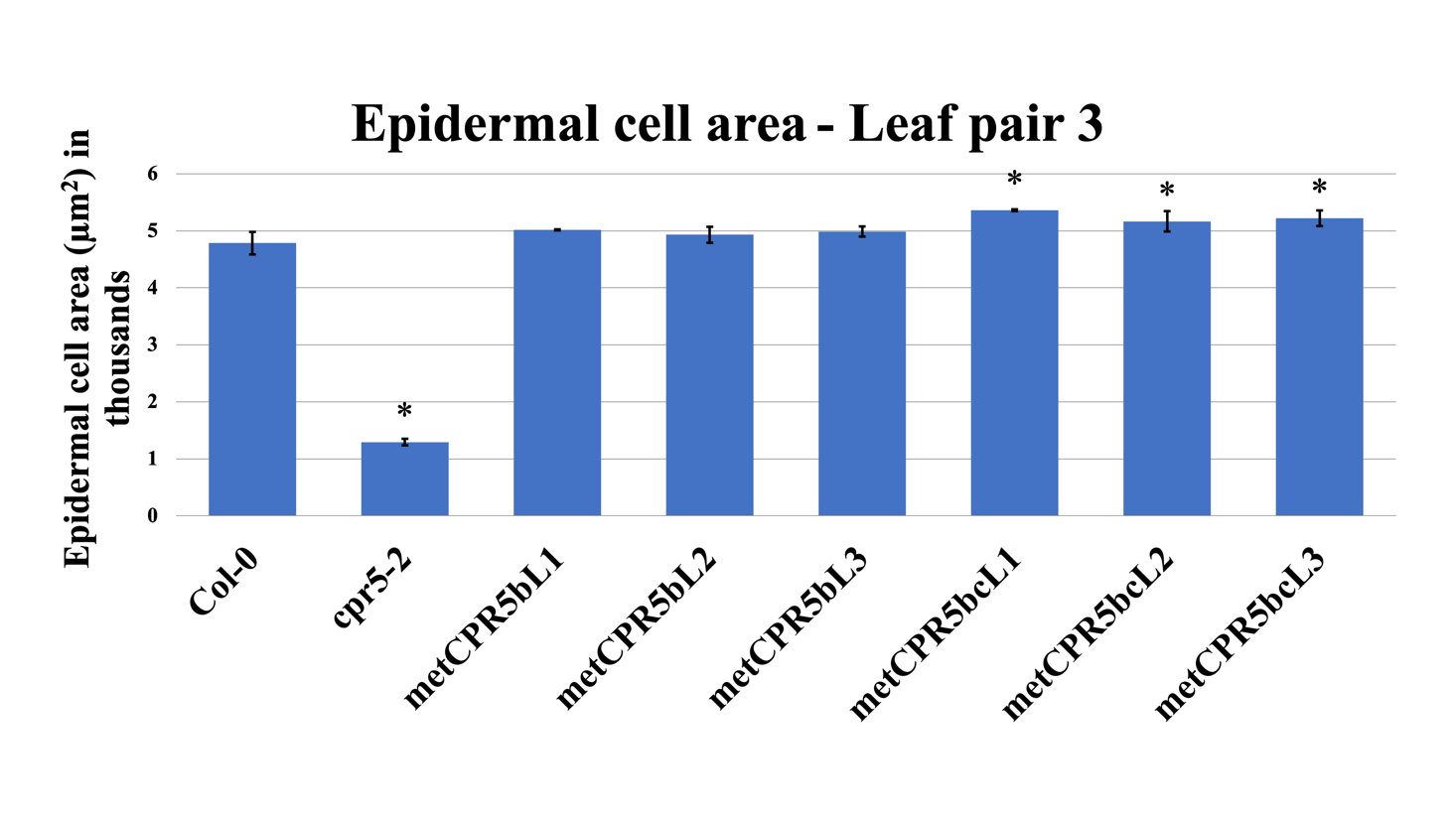


**Figure S3 Area of abaxial epidermal pavement cells.** Leaf 3 was imaged using a scanning electron microscope (SEM). The images were processed and cell areas were measured individually by ImageJ (https://imagej.nih.gov/ij/). Each value is the average of values from three SEM images taken from three leaves. Each of the leaves had 24-26 cells with a complete boundary in the case of Col-0 and *metCPR5*; for *cpr5*, ~150 cells/image were analysed. Error bars represent the standard error, and asterisks indicate significant difference from Col-0 at p < 0.05 (Student´s *t*-test).

**Table S1: List of primers**

| Primer name | Forward strand sequence | Reverse strand sequence |
| --- | --- | --- |
| CPR5 | GGTGATGGCAAACTCTGGCTAATAGTTCG | AAAGATGGCCTCCTCGTCTCAAGCA |
| SynCPR5 | ACTTCCTCAATAAGCGCTCGAGT | ATTAATGGGCCCGAACATCAAT |
| cpr5-2 | GGTGATGGCAAACTCTGGCTAATAGTTCG | CCCACCATGAAGGTGATACATAGCGAAG |
| PR1 | ACACGTGCAATGGAGTTTGTGG | TTGGCACATCCGAGTCTCACTG |
| ACT2 | TCTTCCGCTCTTTCTTTCCAAGC | ACCATTGTCACACACGATTGGTTG |
| UBC9 | TCACAATTTCCAAGGTGCTGC | TCATCTGGGTTTGGATCCGT |
